# Reports on the Hospitals of the United Kingdom

**Published:** 1910-06-25

**Authors:** Henry Burdett


### REPORTS

ON THE

### Hospitals of the United Kingdom.

By SIR HENRY BURDETT, K.C.B., K.C.V.O.

### SOME SMALLER HOSPITALS.

### LII.—ACTON COTTAGE HOSPITAL.

(Known as "The Passmore Edwards Jubilee Cottage Hospital, Nursing Institute, and Invalid Kitchen.")

This institution is not only a hospital, but a nursing institution for the supply of nurses, and an invalid kitchen for the supply of food to the poor of the district. It gives one the impression of being indifferently managed, and the buildings, from a hygienic and architectural point of view, are contemptible for hospital purposes. The buildings, as they originally stood, though unsatisfactory in the sense that many of the windows were more suitable from their dimensions for a prison than for a hospital, that one end of both the men's and women's wards was a blank wall, and that the plan displays an absence of knowledge of hospital requirements, might have passed as an example of much it is desirable to avoid in building a cottage hospital. More than one alteration has been made to the plan, which has taken the form mainly of additions to the blank walls at the end of the old buildings and wards, and each addition has tended to make the institution less fitted to fulfil the requirements of a modern hospital. It represents a type of building which has been put up under circumstances which have not proved favourable to efficiency. Into the buildings as they stand twenty beds and four cots have been crowded, but the average number occupied is fortunately not more than about fourteen. Two stories upstairs are given up to the accommodation of nurses, of whom there were said to be seven on the staff, in addition to the matron, but one of these was the district nurse. Two small wards are given up to the accommodation of private patients, who pay two guineas a week.

Looking at the hospital as we found it, we formed the conclusion that more attention must be paid to the supply of nurses, and the provision of dinners for "invalids outside who are unable to procure special food in ill health," than to the ordinary work of a hospital. It is a great pity, in our judgment, that a suburb of London should have an institution of this kind in its present condition, both in regard to buildings and generally. This hospital should prove an object lesson to persons of wealth who have a sympathy with hospitals and an inclination to build a small one themselves. The advancements in science, the contiguity of the great city, with all its knowledge and hospital and expert facilities in matters relating to the relief of sickness and accident, combined with the difficulty and cost entailed by setting up small district hospitals like this one, in such circumstances, in a metropolitan or indeed any district of a great city, to say nothing of the practical impossibility of guaranteeing the continuous and smart efficiency of the administration of such institutions, all these make it desirable in the interests of the poor, as well as of the capitalists who provide the money, to avoid experiments of the kind. Cottage hospitals were intended for, and should be confined to, country and small urban districts, where they do meet many needs of such a population. They at the same time contribute to the efficiency of the medical service in their immediate neighbourhood. These requirements and advantages are practically confined to rural as opposed to urban communities. Mr. Albert Napper, in starting the cottage hospital movement, designed it for this purpose, and the experience of fifty years in the working of this institution shows that Mr. Napper was right, and that for urban purposes it is far better, and even necessary, to provide completely equipped hospitals of substantial size, where a consulting medical staff is appointed, and where every case can be treated immediately and thoroughly.

### LIII.—THE ALEXANDRA HOSPITAL FOR CHILDREN WITH HIP DISEASE, CLANDON (SURREY) BRANCH.

This branch hospital contains twenty cots, and is situated about two miles from Leatherhead station. The staff consists of the Matron, Miss H. M. Thorold, one staff nurse, and three probationers, one of whom pays. When we inspected it one bright September day it had a very attractive appearance, and the majority of the children were lying in cots in the verandah, where they can stay all night. A little further verandah accommodation would seem to be desirable to enable the whole of the cots to be put outside.

The hospital is well kept, well found, and in good order throughout. The children were contented and happy, and appeared to be doing very well. One of them had been here for five and another for three years.

### LIV.—CREWKERNE HOSPITAL.

This hospital was established in 1866, a new building, the present one, having been opened in 1894. It contains fifteen beds and three cots. The Matron is Miss R. A. Pugh, who has two nurses to assist her.

This hospital is pleasantly situated and presents an attractive exterior. Internally we found it reasonably well kept and the patients comfortable. The wards were airy and inviting.

The Matron was using the pay-ward for her bedroom, a practice which is indefensible hygienically and administratively. No committee is justified in permitting a ward or wards built for the reception of patients to be occupied by an officer or anyone but a patient. Indeed, in our view, no officer in responsible charge of a hospital who has a high sense of duty should ever consent to so grave an abuse as that we have regretfully to call public notice to and to condemn unreservedly.

